# Med-Tech Industry Entry Strategy Analysis under COVID-19 Impact

**DOI:** 10.3390/healthcare8040431

**Published:** 2020-10-25

**Authors:** I-Ching Fang, Peng-Ting Chen, Hsin-Hui Chiu, Chia-Li Lin, Fong-Chin Su

**Affiliations:** 1 Department of Biomedical Engineering, National Cheng Kung University, Tainan 701, Taiwan; dr.icfang@gmail.com (I.-C.F.); chen@ncku.edu.tw (P.-T.C.); 2 Medical Device Innovation Center, National Cheng Kung University, Tainan 701, Taiwan; 3 Institute of International Business, National Cheng Kung University, Tainan 701, Taiwan; nature7772921@gmail.com; 4 Graduate Institute of Adult Education, National Kaohsiung Normal University, Kaohsiung 802, Taiwan; linchiali0704@yahoo.com.tw

**Keywords:** medical technology (Med-Tech), industry entry criteria, COVID-19, IAA-NRM, DEMATEL

## Abstract

COVID-19 has been impacting the Med-Tech industry dramatically since the beginning of 2020. Along with the pandemic continuously growing, the demand for major global medical products such as masks and protective clothing has surged. The Med-Tech industry is facing the huge challenge of a lack of production capacity, including raw material, production equipment, production line, professional human resources, and more. It would require not only the operators in the Med-Tech industry to enlarge their productivity, but also new investors from outside. This study focused on the entry strategy analysis of the Med-Tech industry, developing five driving factors, and conducting an opinion survey from three different aspects, including vendors, channels, and end-users, under COVID-19 impact. A total of 99 valid questionnaires were collected. After that, the Importance Accessibility Analysis-Network Relation Map (IAA-NRM) approach was used to verify the importance and implementation priority of the entry strategies. Then, the Decision Making Trial and Evaluation Laboratory (DEMATEL) technique is used to construct the NRM method. The research results showed that there is a common strategic path, from the regulatory system to operation resources and then marketing promotion. In addition, in these three viewpoints, vendors and end-users have similar priorities in terms of industry attributes and barriers to entry.

## 1. Introduction

Due to the global infectious disease outbreak of COVID-19, the demand for major global medical products such as masks and protective clothing has surged. Based on the presented evidence, the World Health Organization (WHO) has reported that the major transmission route of the COVID-19 virus is through respiratory droplets. That means direct close contact between people should be isolated. Moreover, indirect transmission routes could be contacted in the environment of the infected people, including stuff used by them, such as thermometer, stethoscope, or other medical appliances. In addition, airborne transmission of generated aerosols is the other route. Understanding the nature of the virus and how it spreads is very important to determine what type of protective equipment should be used. It also can help to manage the supplies amid rushing global demand, panic procurement, trade restrictions, misinformation, and other factors [1].

There is a shortage of personal protective equipment around the world, such as surgical masks, goggles, full-body protective clothing, N95 masks, and more. A critical component for combatting the COVID-19 pandemic is to have the right diagnostic tests available [2]. At the same time, WHO estimates that the global supply of personal protective equipment needs to increase by 40%. After the outbreak of COVID-19, Taiwan has faced insufficient medical materials like other countries in the world. Since, in addition to the basic needs of general medical institutions, Med-Ted enterprises do not stock large amounts of spare material or parts unless there are major infectious diseases. Therefore, when a major epidemic breaks out, the obvious result would be a huge lack of medical supplies. The Taiwan government has temporarily banned the export of important medical materials such as masks and reduced the stock of distributors through centralized recruitment. Then, in order to distribute masks to people, the government formed a national mask production team by recruiting medical manufacturers as an industrial alliance to control all mask production. All the production, distribution, and sales are well planned by the government. On the other hand, medical material manufacturers in China have improved their production capacity by simplifying the equipment procurement process and improving labor assistance. After domestic demand is met, the excess capacity will be gradually exported to other countries in need. This sudden COVID-19 epidemic allows the government to re-examine the development of the medical material industry, and it also drives a new wave of demand in the medical industry.

Large manufacturers have dominated the Med-Tech industry all over the world. In the United States, pure Med-Tech companies account for 90% of medical technology revenue and 86% of the total market value. They make up 16% of the listed enterprises in the United States. In addition, the top 18 Med-Tech companies in Europe contributed 89% of revenue and 90% of the market value [3]. With innovative and high technology products, the US Med-Tech companies are in a leading position in the world. The nature of the Med-Tech industry is the R&D budgets accounting for a large part of company expenditures, on an average of 7% of revenue. However, more than 80% of Med-Tech companies have fewer than 50 employees in the United States [4]. In 2013, the Med-Tech market reached approximately US $4.3 billion in Taiwan. The compound annual growth rate was about 8% between 2008 and 2013. Moreover, there are more than 700 Med-Tech companies in Taiwan. Most of their products are middle or low-end medical equipment [5]. Most of Taiwan’s Med-Tech enterprises are small manufacturers. Still, the products are sold all over the world.

Since the COVID-19 pandemic, the surge in demand for medical supplies has given the Med-Tech industry more attention. Recently, large-scale mergers and acquisitions in the Med-Tech industry have emerged one after another, and the annual sales growth rate has repeatedly reached new highs, which once again proves that Med-Tech is another forward-looking industry in the future. However, the industrial characteristics of Med-tech are completely different from other industries. All the stakeholders in the Med-Tech industry chain are restricted by strict licenses or laws and regulations. The end-users of Med-Tech products, instead of regular people, are professional customers, such as doctors and pharmacists. In addition, most equipment needs special training in order to install and use it. Therefore, when Med-Tech products enter the market, they need to undergo special training. Not all users can buy goods at will. Therefore, in order to enter the Med-Tech industry, all relevant stakeholders, such as end-users, sales channels, and manufacturers, need to be investigated. Finding the common path of these stakeholders is regarded as a key factor for consideration. There are still small numbers of studies on the practical process of entering the Med-Tech industry. This study focused on strategies of new capabilities to adapt to the new environment for companies to enter the Med-Tech industry from three stakeholders’ perspectives. 

## 2. Materials and Methods

The modified Importance Accessibility Analysis-Network Relation Map (IAA-NRM) approach was adopted to analyze the Med-Tech industry entry strategies. First, we determined the driving factors (aspects/criteria) of the industrial entry consideration through a literature review and expert interviews. Then, we surveyed the importance level and accessibility level for each aspect/criterion and adopted the IAA approach to the status of importance and accessibility in the second stage. After that, this study analyzed the network relation structure for the industrial entry strategies based on the NRM approach and determined the dominant aspects/criteria. Finally, the study combined the results of the IAA approach and the NRM approach to propose a development strategy for Med-Tech industrial entry criteria. 

### 2.1. The Expert Interviews

After analyzing literature and related works, this study conducted expert interviews to gain the driving factors. There were 18 experts involved in this study, including five Med-Tech international enterprise owners, three government agencies or Med-Tech association managers, two experienced professors who were involved in practical projects, two Med-Tech vice presidents with R&D specialty, two Med-Tech vice presidents with a sales and marketing specialty, and four doctors and pharmacists. They are all experienced experts in Taiwan. Table 1 showed the results from expert interviews. There are five aspects, including Professional Capability (PC), Business Management (MB), Operation Resources (OR), Regulation System (RS), and Marketing Promotion (MP). Each aspect contained four criteria as the descriptions in Table 1. 

### 2.2. The Questionnaire Design and the Reliability Analysis

This study consulted stakeholders for aspects/criteria of a potential questionnaire and then designed the stakeholders’ surveys accordingly. This study aggregates the stakeholders’ interview results and generalizes five evaluation aspects and twenty criteria. The five aspects were Professional Capability, Business Management, Operation Resources, Regulation System, and Marketing Promotion as shown in Table 1. The researcher evaluated the satisfaction and importance status based on the different stakeholders, which were vendors, channels, and end users, through the paper and online questionnaire. This study collected 150 samples. After deducting samples that did not belong to the three groups and questionnaires with all the same values, 99 valid samples were collected from three groups, including 55 vendors, 17 channels, and 27 end users. The reliability of the importance index and accessibility index was based on the Cronbach alpha value calculation. The reliability of the importance index was 0.931, and the reliability of the accessibility index was 0.907. The reliability of the importance index and accessibility index was higher than the suggested reliability level of 0.7, which means the importance index and accessibility index were highly consistent. The reliability of the aspects of the evaluation system was 0.921, also indicating that the aspect was highly consistent as shown in Table 2.

### 2.3. The IAA Approach

Scholars have proposed evaluation models similar to the IAA approach to analyze the important relationships and priorities among factors, such as Lin, C. [6] proposed an Innovation Opportunity Analysis-Network Relation Map (IOA-NRM) approach to analyze the value-driving forces for digital transformation for the tourism enterprise strategy decision. Moreover, Wang, Lin, Chia, Chung, and Lee [7] developed a Satisfied Importance Analysis (SIA) to evaluate the performance of each division. In addition, they proposed an evaluation laboratory technique, Decision Making Trial and Evaluation Laboratory (DEMATEL), to catch the causal relationships among factors and produce an influence-relationship map. Furthermore, Wang, Lin, Wang, Liu, and Lee [8] developed an Importance-Satisfaction Analysis (ISA) model to estimate the performance of each factor and an Influence-Relations Map (IRM) to catch the relationship among factors.

**Figure 1:**
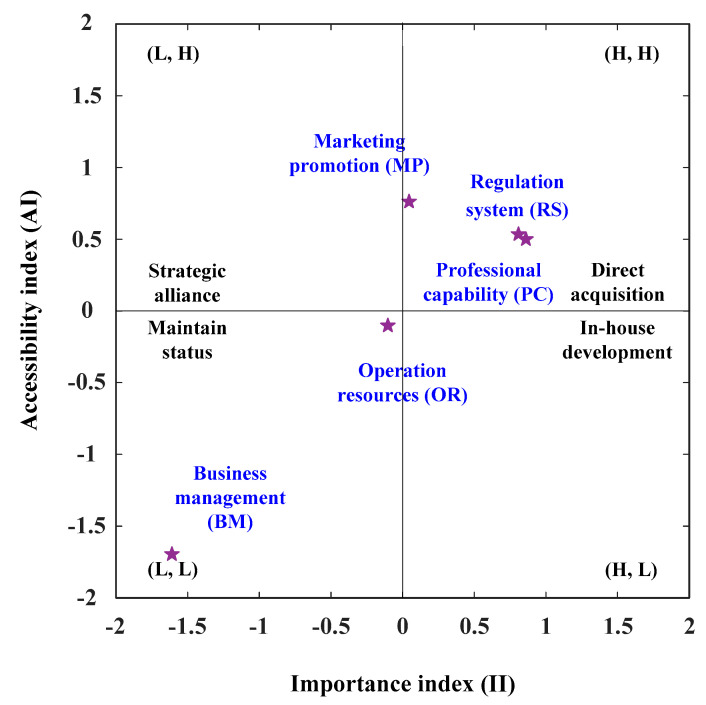
The analysis map of IAA (Importance Accessibility Analysis).

Analysis of the degree of importance and accessibility of criteria was conducted, and the surveyed data were normalized into equal measuring scales. According to the survey data results, the criteria are divided into four categories: The first quadrant is a high degree of importance with a high degree of accessibility, shown by the symbol (H, H). The second quadrant of criteria is a low degree of importance level with a high degree of accessibility level, indicated by the symbol (L, H). The third quadrant of criteria is a low degree of importance with a low degree of accessibility, shown by the symbol (L, L). The fourth quadrant of criteria is a high degree of importance with a low degree of accessibility, indicated by the symbol (H, L). In this study, the analysis of IAA is as follows: The first step is to improve those aspects (i.e., Regulation System, Professional Capability, and Marketing Promotion) falling into the first quadrant (H, H). The aspects/criteria of the first quadrant (H, H) were high importance level and high accessibility level in the IAA analysis. The second step is to improve those aspects (i.e., Business Management and Operation Resources) falling into the third quadrant (L, L). Because, the aspects/criteria of the third quadrant (L, L) was a low importance level and low accessibility level as shown in Table 3 and Figure 1.

### 2.4. The NRM Analysis Based on the DEMATEL Technique

The DEMATEL technique is used to construct the Network Relation Map (NRM) for the industrial entry analysis. When users are making decisions about industrial entry, there are many criteria they may consider. The most common problem they face is that these criteria impact each other. Therefore, before making developments to criteria, it is necessary to know the basic criteria and then make useful developments to enhance overall satisfaction. When a decision-maker needs to improve many criteria, the best way to handle this is to determine the criteria that most impact others and improve them. 

Some recent studies have adopted the DEMATEL approach for evaluating complex problems, such as the user interface analysis based on the DEMATEL approach [9], failure sorting evaluation system [10], the innovation policy portfolios of Taiwan’s SIP Mall industry, based on the DEMAEL approach [11], the causal analytical method of group decision-making, based on the fuzzy DEMATEL approach [12], the evaluation of an airline safety management system [13], the analysis of value-created systems for science (technology) parks [14], the service selection of vehicle telematics system [15], the integrated model of relation structure analysis and performance evaluation for hot spring hotels, based on the hybrid Multiple Criteria Decision Making (MCDM) technique [16], the integrated approach of selection of strategic alliance partners for the airline industry [17], the selection model of outsourcing provider for the airline industry [18], the evaluation of supply chain performance based on the DEA (Data Envelopment Analysis) and balanced scorecard technique [19], the portfolio selection of strategic project for national research institutes based on the hybrid MCDM method [20], the quality education service for Hospitality, Tourism, and Leisure Undergraduate Programs (HTLPs) [21], the critical factors of implementing a program for international Multivariate Imputation via Chained Equations (MICE) professionals, based on the hybrid MCDM model [22], determining the causal relationships of the antecedents of organizational citizenship behavior (OCB) for the hospitality industry, using the fuzzy DEMATEL [23], the service position model of package tour services by the hybrid MCDM approach [24], the sustainable development strategies of industrial tourism, based on the IOA-NRM approach [6], and the blockchain critical success factors of a sustainable supply chain [25]. This study divides DEMATEL into five steps: (1) calculate the original average matrix, (2) calculate the direct influence matrix, (3) calculate the indirect influence matrix, (4) calculate the full influence matrix, and (5) analyze the NRM (Network Relation Map). 

(1) Calculate the original average matrix

Respondents were asked to indicate the influence that they believed each aspect exerts on each of the others, according to a scoring scale ranging from 0 to 4. “0” means no influence, whereas “4” means “extreme influence” between aspect/criterion. “1”, “2”, and “3” mean “low influence”, “medium influence”, and “high influence”, respectively. As shown in Table 4, the influence that “Operation Resources (OR)” has on “Professional Capability (PC)” is 3.030, which means “high influence”. The influence that “Professional Capability (PC)” has on “Business Management (BM)” is 2.727, which also means “medium influence”, as shown in Table 4. 

(2) Calculate the direct influence matrix

From Table 4, we processed the “Original average influence matrix” (*A*) by Equations (1) and (2) and got the “Direct influence matrix” (*D*). As shown in Table 5, the diagonal items of *D* are all 0, and the sum of a row is at most 1. Then, we developed Table 5 by adding up rows and columns. In Table 6, the sum of rows and columns for “Business Management (BM)” is 1.975, which is the most important influence aspect. On the other hand, the sum of row and column for “Regulation System (RS)” is 1.886, which is the least essential influence aspect:

(1) D=sA, s>0,

where

(2) $$s=\underset{i,j}{\min}\;[1/\underset{1\leq i\leq n}{\max}\sum_{j=1}^{n}a_{ij},1/\underset{1\leq j\leq n}{\max}\sum_{i=1}^{n}a_{ij}],i,j=1,2,\ldots ,n,$$

and $\underset{m\to \infty }{\lim}\;D^{m}={\left[0\right]}_{n\times n}$, where $D=[x_{ij}{]}_{n\times n}$, when $0<\sum_{j=1}^{n}x_{ij}\leq 1\;$ or $0<\sum_{i=1}^{n}x_{ij}\leq 1$, and at least one $\sum_{j=1}^{n}x_{ij}$ or $\sum_{i=1}^{n}x_{ij}$ equals one, but not all. So, we can guarantee $\underset{m\to \infty }{\lim}\;D^{m}={\left[0\right]}_{n\times n}$.

(3) Calculate indirect influence matrix

The indirect influence matrix can be derived from Equation (3), as shown in Table 7:

(3) $$ID=\sum_{i=2}^{\infty }D^{i}=D^{2}{\left(I-D\right)}^{-1}.$$

(4) Calculate the full influence matrix

Full influence matrix ***T*** can be derived from Equations (4) or (5); Table 8 is the calculated full influence matrix T. As shown in Table 8, the full influence matrix T consists of multiple elements, as indicated in Equation (6). The sum vector of row value is {d}, and the sum vector of column value is; the sum vector of row value plus column value is {di + ri}, which describes the full influence of matrix T. As the sum of row value plus column value {di + ri} is higher, the correlation of the dimension or criterion is stronger. The sum of row value minus column value is {di − ri}, which describes the net influence relationship. If di − ri > 0, it means the degree of influencing others is stronger than the degree of being influenced. As shown in Table 9, the BM (Business Management) aspect was the highest degree of full influence ($d_{2}$ + $r_{2}$ = 75.289). The RS (Regulation System) aspect is the highest net influence ($d_{4}$ − $r_{4}$ = 1.195). The order of other net influences is listed as follows: Operation Resources (OR) ($d_{3}$ − $r_{3}$ = 0.849), Business Management (BM) ($d_{2}$ − $r_{2}$ = −0.029), Professional Capability (PC) ($d_{1}$ − $r_{1}$ = −0.646), and Marketing Promotion (MP) ($d_{5}$ − $r_{5}$ = −1.369):

(4) $$T=D+ID=\sum_{i=1}^{\infty }D^{i},$$

(5) $$T=\sum_{i=1}^{\infty }D^{i}=D{\left(I-D\right)}^{-1},$$

(6) $$T=[t_{ij}],\;i,j\in \{1,2,\ldots ,n\},$$

(7) $$d=d_{n\times 1}={\left[\sum_{j=1}^{n}t_{ij}\right]}_{n\times 1}=(d_{1},\ldots d_{i},d_{n}),$$

(8) $$r=r_{n\times 1}={[\sum_{i=1}^{n}t_{ij}{]}^{\prime }}_{1\times n}=(r_{1},\ldots r_{j},\ldots r_{n}).$$

(5) Analyze the NRM (Network Relation Map)

Experts were invited to discuss the relationships and influence levels of criteria under the same aspects/criteria defined in Table 1, and to score the involvement and influence among criteria based on the DEMATEL approach. Aspects/criteria were divided into different types, so the experts could answer the questionnaire in areas/fields with which they were familiar. The net full influence matrix, $C_{net}$, is determined by Equation (9):

(9) $$C_{net}=[t_{ij}-t_{ji}],\;i,j\in \{1,2,\ldots ,n\}.$$

**Figure 2:**
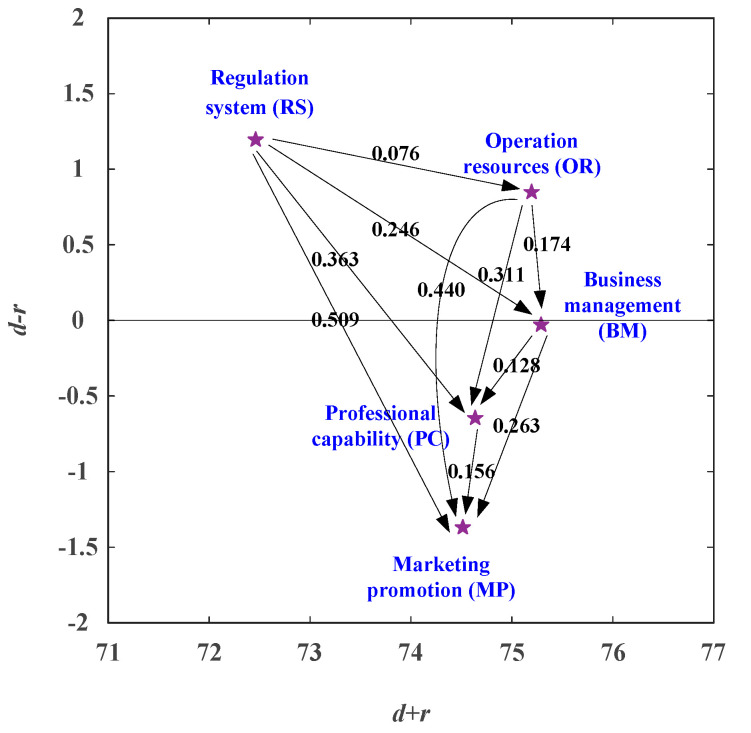
The NRM (Network Relation Map) for urban sustainable development.

The diagonal items of the matrix are all 0. In other words, the matrix contains a strictly upper triangular matrix and a strictly lower triangular matrix. Moreover, while the values of the strictly upper and strictly lower triangular matrix are the same, their symbols are opposite. This property helps us; we only have to choose one of the strictly triangular matrices. Table 8 shows the full influence matrix, and Equation (9) can get the net influence matrix shown in Table 10. Using the values of (d + r) and (d − r) in Table 9 as X and Y values, respectively, the NRM approach can be drawn, as shown in Figure 2 [26]. Figure 2 indicates that Regulation System (RS) aspect is the primary aspect with net influence, while Marketing Promotion (MP) aspect is the primary aspect being influenced. The aspect of Business Management (BM) is the aspects with the highest full influence, while the Regulation System (RS) is the one with the smallest full influence aspect. 

### 2.5. The IAA-NRM Approach

The IAA-NRM (Importance Accessibility Analysis-Network Relation Map) analytic process includes two stages. The first step is the IAA approach, and the second step is the NRM analysis approach. The IAA analysis determines the aspects/criteria status of satisfaction and importance degree for industrial entry; the IAA analysis can help decision-makers identify criteria that should be developed when the standard satisfied degree is less than the average satisfied degree. The four development strategies are presented in Table 11. Development strategy A (direct acquisition) can apply to the aspects of RS (Regulation System), PC (Professional Capability), and MP (Marketing Promotion). Development strategy C (maintain status) can apply to the aspects of OR (Operation Resources) and BM (Business Management). 

**Figure 3:**
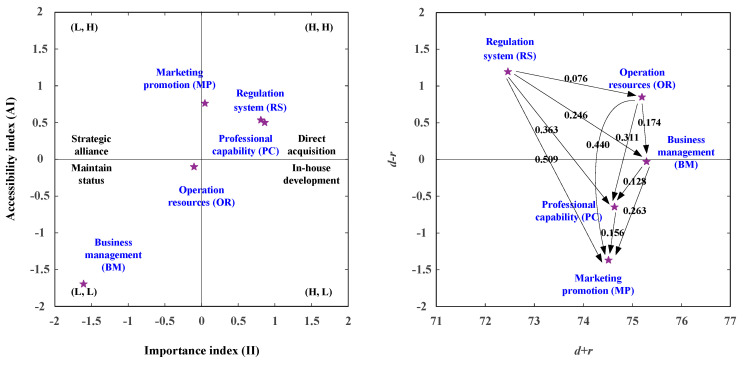
The improvement strategy map of industrial entry.

The IAA-NRM approach determines the aspects/criteria that should be developed using IAA analysis, and the development path based on the NRM approach. We can determine that the aspects of RS (Regulation System), PC (Professional Capability), and MP (Marketing Promotion) should be developed, and the RS (Regulation System) is the aspect that is the primary aspect with net influence, as shown in Figure 3. So, we can improve the Marketing Promotion (MP) aspect by addressing the RS, OR, BM, and PC aspect. The MP aspect is the primary aspect being influenced; therefore, the aspect of MP can be developed by the aspects of RS, OR, BM, and PC as shown in Figure 3 and Table 11.

### 2.6. Evaluation the Suited Development Paths Using the Aspects/Criteria Rank

In the analysis of the suited development path, the ranking of the II (Importance Index) is PC⊃RS⊃MP⊃OR⊃BM and the ranking of the AI (Accessibility Index) is MP⊃RS⊃PC⊃OR⊃BM, as shown in Table 12. The eight development paths (RS→MP; RS→OR→MP; RS→BM→MP; RS→PC→MP; RS→OR→PC→MP; RS→OR→BM→MP; RS→BM→PC→MP; RS→OR→BM→PC→MP) can be determined through the NRM approach, then the disadvantage aspects/criteria can be improved by the advantage aspects/criteria. The underline paths represented the effective paths.

The II (Importance Index) ranking is PC⊃RS⊃MP⊃OR⊃BM, and the MP (Marketing Promotion) aspect can be improved through the RS (Regulation System) aspect in the first development path (RS [2]→MP [3]). The OR (Operation Resources) aspect can be developed through the RS aspect in the second development path (RS[2]→OR[4]→MP[3]). Then, the BM (Business Management) aspect can be improved through the RS aspect in the third development path (RS[2]→BM[5]→MP[3]). The MP aspect can be improved by the PC (Professional Capability) aspect in the fourth development path (RS[2]→PC[1]→MP[3]), as shown in Table 12. The OR aspect can be improved through the RS aspect, and the MP aspect can be developed by the PC aspect in the five development path (RS[2]→OR[4]→PC[1]→MP[3]). Then, the BM aspect can be improved through the OR aspect, and the OR aspect can be developed by the RS aspect of the sixth development path (RS[2]→OR[4]→BM[5]→MP[3]). The BM aspect can be developed by the RS aspect, and the MP aspect can be developed through the PC aspect in the seven development path (RS[2]→BM[5]→PC[1]→MP[3]). The BM aspect can be developed by the OR aspect, and the OR aspect can be developed through the RS aspect, and then the MP aspect can be developed through the PC aspect in the fourth development path (RS[2]→OR[4]→BM[5]→PC[1]→MP[3]), as shown in Table 12.

The AI (Accessibility Index) ranking is MP⊃RS⊃PC⊃OR⊃BM, and the MP (Marketing Promotion) aspect cannot be developed through the RS (Regulation System) aspect in the first development path (RS[2]→MP[1]). The OR aspect can be developed by the RS aspect in the second development path (RS[2]→OR[4]→MP[1]). Then, the BM (Business Management) aspect can be developed through the RS aspect in the third development path (RS[2]→BM[5]→MP[1]). The MP aspect can be developed by the PC (Professional Capability) aspect in the fourth development path (RS[2]→PC[3]→MP[1]), as shown in Table 12. The OR aspect can be developed through the RS aspect, and the MP aspect can be developed by the PC aspect in the five development path (RS[2]→OR[4]→PC[3]→MP[1]). Then, the BM aspect can be developed through the OR aspect, and the OR aspect can be developed by the RS aspect of the sixth development path (RS[2]→OR[4]→BM[5]→MP[1]). The BM aspect can be developed by the RS aspect, and the MP aspect can be developed through the PC aspect in the seven development path (RS[2]→BM[5→PC[3]→MP[1]). The BM aspect can be developed by the OR aspect, and the OR aspect can be developed through the RS aspect, and then the MP aspect can be developed through the PC aspect in the fourth development path (RS[2]→OR[4]→BM[5]→PC[3]→MP[1]), as shown in Table 12**.** This study combines the development paths of II (Importance Index) and AI (Accessibility Index) and determine the seven improvement paths (RS→OR→MP; RS→BM→MP; RS→PC→MP; RS→OR→PC→MP; RS→OR→BM→MP; RS→BM→PC→MP; RS→OR→BM→PC→MP) as shown in Table 12.

## 3. Results

This study adopts the empirical case of industrial entry to present the IAA-NRM approach. We investigated the degree of satisfaction and importance of the questionnaire. This study uses the IAA technique to evaluate the status of importance and accessibility and adopt the NRM technique to determine the network relation structure. Besides, the study also combines the IAA technique and NRM technique to determine the development strategy and suited development paths for the industrial entry. This sub-section introduces the IAA-NRM approach of four aspects for industrial entry. The IAA determines the aspects/criteria that should be developed; the NRM determines the development strategy and suited improvement paths. The IAA-NRM analysis of the IAA approach and NRM approach can present the development strategy and common suited development path for industrial entry.

### 3.1. The Suited Development Paths for Vendors

**Figure 4:**
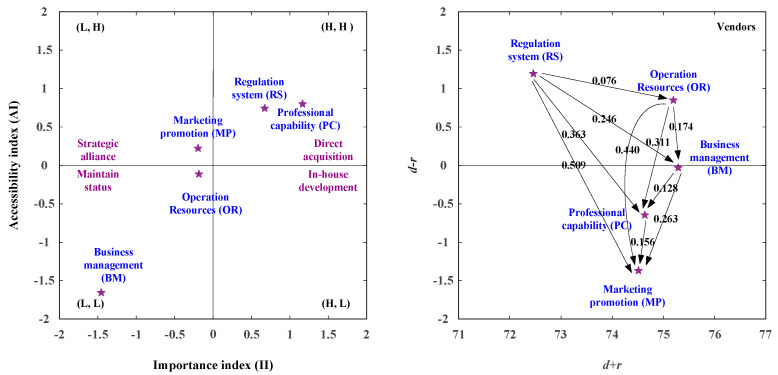
The Importance Accessibility Analysis-Network Relation Map (IAA-NRM) for vendors.

In the vendors’ viewpoint, this study illustrates the development strategy map based on the IAA-NRM approach, as shown in Figure 4 and Table 13, and integrates the IAA technique and the NRM technique, as illustrated in Table 13. Then, the net influence matrix for residents is shown in Table 13. In the analysis of the IAA, the RS (Regulation System) aspect and PC (Professional Capability) aspect are the importance degrees more than the average importance degree and the accessibility degree is also more than the average accessibility degree. Consequently, the two aspects should be developed, while the importance degree of the aspects increases more than the average importance degree (II > 0), as illustrated in Figure 4 and Table 13.

In the analysis of NRM, the aspects of RS and OR are the positive net influence effect (*d* − *r* > 0), so they can improve the RS aspect by itself as shown in Figure 4 and Table 13. The three development strategies were present in Table 13. Development strategy A (direct acquisition) can apply to the aspects of RS and PC. Development strategy B (strategic alliance) can apply to the MP aspect and the Development strategy C (maintain status) can apply the aspects of OR and BM. As shown in Table 13 and Figure 4, the aspects of RS and PC were located in the first quadrant [(H, H)], so the two aspects should be enhanced. The PC aspect can be developed by the aspects of RS, OR, and BM. The RS aspect can only be developed by itself, as illustrated in Figure 4 and Table 13.

### 3.2. The Suited Development Paths for Channels

**Figure 5:**
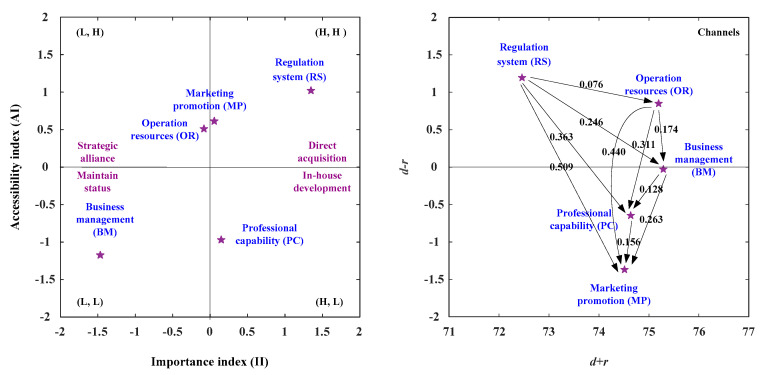
The NRM for channels.

In the channels’ viewpoint, this study illustrates the development strategy map based on the IAA-NRM approach, as shown in Figure 5 and Table 14, and integrates the IAA approach and the NRM approach, as illustrated in Table 14. The net influence matrix for channels is shown in Table 14. In the analysis of the IAA, the aspects of RS (Regulation System) and MP (Marketing Promotion) are the II (Importance Index) more than the average importance degree (II > 0) and the AI (Accessibility Index) is also more than the average accessibility degree (AI > 0). Consequently, the two aspects should be enhanced as illustrated in Figure 5 and Table 14. In the analysis of NRM, the aspects of RS (Regulation System) and OR (Operation Resources) constitute the positive net influence effect (*d* − *r* > 0), so they can influence the aspect of MP (Marketing Promotion) through the aspects of RS (Regulation System), OR (Operation Resources), BM (Business Management), and PC (Professional Capability). The four development strategies were present in Figure 5 and Table 14. Development strategy A (direct acquisition) can apply to the RS (Regulation System) aspect and MP (Marketing Promotion). Development strategy B (strategic alliance) can apply to the OR (Operation Resources) aspect and the development strategy C (maintain status) can apply to the BM (Business Management). The development strategy D (in house development) can apply to the PC (Professional Capability) aspect. The aspects of RS (Regulation System) and MP (Marketing Promotion) were located in the first quadrant [(H, H)], so the two aspects should be enhanced. The MP (Marketing Promotion) aspect can be developed by the aspects of RS, OR, BM, and PC. The RS (Regulation System) aspect can only be developed by itself, as illustrated in Figure 5 and Table 14.

### 3.3. The Suited Development Paths for End Users

**Figure 6:**
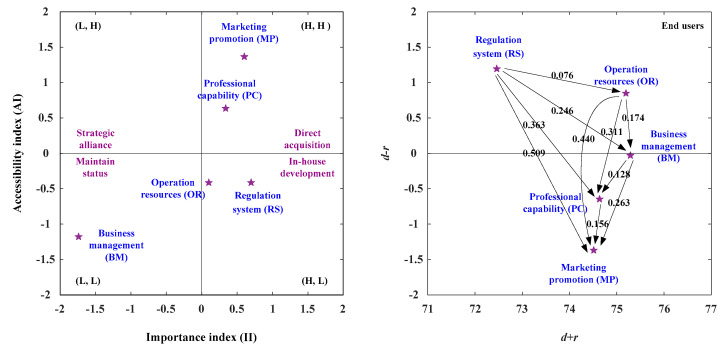
The IAA-NRM for end users.

In the end users’ viewpoint, this study illustrates the development strategy map based on the IAA-NRM approach and integrates the IAA approach and the NRM approach, as illustrated in Table 15. The net influence matrix for end users is shown in Table 15. In the analysis of the IAA, the aspects of MP (Marketing Promotion) and PC (Professional Capability) are the II (Importance Index) more than the average importance degree (II > 0) and the AI (Accessibility Index) is also more than the average accessibility degree (AI > 0). Consequently, the two aspects should be enhanced as illustrated in Figure 6 and Table 15.

In the analysis of NRM, the aspects of RS and OR constitute the positive net influence effect (*d* − *r* > 0), so they can influence the MP aspect by the aspects of RS, OR, BM, and PC. Besides, they also influence the PC aspect through the RS, OR, and BM. The four development strategies were present in Figure 6 and Table 15. Development strategy A (direct acquisition) can apply to the aspects of PC and MP. The development strategy C (maintain status) can apply to the BM (Business Management), and the development strategy D (in house development) can apply to the aspects of OR and RS. The aspects of PC (Professional Capability) and MP (Marketing Promotion) were located in the first quadrant [(H, H)], so the two aspects should be enhanced. The MP aspect can be developed by the aspects of RS, OR, BM, and PC. The PC (Professional Capability) aspect can be strengthen by the aspects of RS, OR, and BM as illustrated in Figure 6 and Table 15.

## 4. Discussion

### 4.1. Vendors

In the suited development path analysis, the ranking of the II (Importance Index) is PC⊃RS⊃OR⊃MP⊃BM and the ranking of the AI (Accessibility Index) is PC⊃RS⊃MP⊃OR⊃BM in the vendors, as shown in Table 16. The eight development paths (RS→MP; RS→OR→MP; RS→BM→MP; RS→PC→MP; RS→OR→PC→MP; RS→OR→BM→MP; RS→BM→PC→MP; RS→OR→BM→PC→MP) can be determined based on the NRM approach, and the disadvantage aspects/criteria can be improved through the advantage aspects/criteria.

The ranking of the II (Importance Index) is PC⊃RS⊃OR⊃MP⊃BM, and the MP (Marketing Promotion) aspect can improved by the RS (Regulation System) aspect in the first development path (RS[2]→MP[4]). The MP aspect can be improved through the OR aspect and OR can be improved through the RS aspect in the second development path (RS[2]→OR[3]→MP[4]), and then the BM aspect can be improved by the RS aspect in the third development path (RS[2]→BM[5]→MP[4]). The MP aspect can be improved by the PC aspect in the fourth development path (RS[2]→PC[1]→MP[4]). The OR aspect can be improved through the RS aspect and MP aspect can be improved through the PC aspect in the fifth development path (RS[2]→OR[3]→PC[1]→MP[4]), and then the BM aspect can be improved by the OR aspect and the OR aspect can be improved by the RS aspect in the sixth development path (RS[2]→OR[3]→BM[5]→MP[4]). The BM aspect can be improved through the RS aspect and MP aspect can be improved through the PC aspect in the seventh development path (RS[2]→BM[5]→PC[1]→MP[4]). The BM aspect can be improved by the OR aspect and OR aspect can be improved by the RS aspect and then the MP aspect can be improved by the PC aspect in the eight development path (RS[2]→OR[3]→BM[5]→PC[1]→MP[4]) as shown in Table 16.

The ranking of the AI (Accessibility Index) is PC⊃RS⊃MP⊃OR⊃BM, and the RS aspect can improve the MP aspect in the first development path (RS[2]→MP[3]). The RS aspect can improve the OR aspect in the second development path (RS[2]→OR[4]→MP[3]), and then the RS aspect can improve the BM aspect in the third development path (RS[2]→BM[5]→MP[3]). The PC aspect can improve the MP aspect in the fourth development path (RS[2]→PC[1]→MP[3]). The RS aspect can improve the OR aspect, and then the PC aspect influence the MP aspect in the fifth development path (RS[2]→OR[4]→PC[1]→MP[3]). The RS aspect can improve OR aspect and the OR aspect can improve the BM aspect in the sixth development path (RS[2]→OR[4]→BM[5]→MP[3]). The RS aspect can improve BM aspect and the PC can improve the MP aspect in the seventh development path (RS[2]→BM[5]→PC[1]→MP[3]). The RS aspect can improve the OR aspect and the OR can improve the BM aspect and then the PC aspect can influence the MP aspect in the eighth development path (RS[2]→OR[4]→BM[5]→PC[1]→MP[3]) as shown in Table 16. Hence, the IAA-NRM approach combines the result of the II (Importance Index) development paths and AI (Accessibility Index) development paths; the suited development paths are shown in Table 16. Because the II (Importance Index) development paths and AI (Accessibility Index) development paths are the same in the empirical result, the suited development paths include the two development paths (RS→MP; RS→OR→MP; RS→BM→MP; RS→PC→MP; RS→OR→PC→MP; RS→OR→BM→MP; RS→BM→PC→MP; RS→OR→BM→PC→MP) for vendors, as shown in Table 16.

### 4.2. Channels

In the suited development path analysis, the ranking of the II (Importance Index) is RS⊃PC⊃MP⊃OR⊃BM and the ranking of the AI (Accessibility Index) is RS⊃ME⊃OR⊃PC⊃BM in the sojourners, as shown in Table 17. The four development paths (RS→MP; RS→OR→MP; RS→BM→MP; RS→PC→MP; RS→OR→PC→MP; RS→OR→BM→MP; RS→BM→PC→MP; RS→OR→BM→PC→MP) can be determined based on the NRM approach, and the disadvantage aspects/criteria can be improved through the advantage aspects/criteria.

The ranking of the II (Importance index) is RS⊃PC⊃MP⊃OR⊃BM, and the MP (Marketing Promotion) aspect can improved by the RS (Regulation System) aspect in the first development path (RS[1]→MP[3]) as shown in Table 17. The OR aspect can be improved through the RS aspect in the second development path (RS[1]→OR[4]→MP[3]), and then the BM aspect can be improved by the RS aspect in the third development path (RS[1]→BM[5]→MP[3]). The PC aspect can be improved the RS aspect in the fourth development path (RS[1]→PC[2]→MP[3]), as shown in Table 17. The OR aspect can be improved through the RS aspect and MP aspect can be improved through the PC aspect in the fifth development path (RS[1]→OR[4]→PC[2]→MP[3]), and then the BM aspect can be improved by the OR aspect and the OR aspect can be improved by the RS aspect in the sixth development path (RS[1]→OR[4]→BM[5]→MP[3]). The BM aspect can be improved through the RS aspect and MP aspect can be improved through the PC aspect in the seventh development path (RS[1]→BM[5]→PC[2]→MP[3]). The BM aspect can be improved by the OR aspect and OR aspect can be improved by the RS aspect and then the MP aspect can be improved by the PC aspect in the eight development path (RS[1]→OR[4]→BM[5]→PC[2]→MP[3]) as shown in Table 17.

The ranking of the AI (Accessibility Index) is RS⊃ME⊃OR⊃PC⊃BM, and the RS aspect can improve the ME aspect in the first development path (RS[1]→ME[2]). The RS aspect can improve the OR aspect in the second development path (RS[1]→OR[3]→ME), and then the RS aspect can improve the OM aspect in the third development path (RS[1]→OM[5]→ME[2]). The RS aspect can improve the PC aspect in the fourth development path (RS[1]→PC[4]→ME[2]). The RS aspect can improve the OR aspect, and then the OR aspect influence the PC aspect in the fifth development path (RS[1]→OR[3]→PC[4]→ME[2]). The RS aspect can improve OR aspect and the OR aspect can improve the OM aspect in the sixth development path (RS[1]→OR[3]→OM[5]→ME[2]). The RS aspect can improve OM aspect in the seventh development path (RS[1]→OM[5]→PC[4]→ME[2]), and then the RS aspect can improve the OR aspect and the OR can improve the OM aspect in the eighth development path (RS[1]→OR[3]→OM[5]→PC[4]→ME[2]) as shown in Table 17. Hence, the IAA-NRM approach combines the result of the II (Importance Index) development paths and AI (Accessibility Index) development paths; the suited development paths are shown in Table 17. Because the II (Importance Index) development paths and AI (Accessibility Index) development paths are the same in the empirical result, the suited development paths include the two development paths (RS→MP; RS→OR→MP; RS→BM→MP; RS→PC→MP; RS→OR→PC→MP; RS→OR→BM→MP; RS→BM→PC→MP; RS→OR→BM→PC→MP) for channels, as shown in Table 17.

### 4.3. End Users

In the suited development path analysis, the ranking of the II (Importance Index) is RS⊃MP⊃PC⊃OR⊃BM and the ranking of the AI (Accessibility Index) is MP⊃PC⊃RS⊃OR⊃BM in the sojourners, as shown in Table 18. The eight development paths (RS→MP; RS→OR→MP; RS→BM→MP; RS→PC→MP; RS→OR→PC→MP; RS→OR→BM→MP; RS→BM→PC→MP; RS→OR→BM→PC→MP) can be determined based on the NRM approach, and the disadvantage aspects/criteria can be improved through the advantage aspects/criteria.

The ranking of the II (Importance Index) is RS⊃PC⊃MP⊃OR⊃BM, and the MP aspect can improved by the RS aspect in the first development path (RS[1]→MP[2]). The OR aspect can be improved through the RS aspect in the second development path (RS[1]→OR[4]→MP[2]), and then the BM aspect can be improved by the RS aspect in the third development path (RS[1]→BM[5]→MP[2]). The PC aspect can be improved through the RS aspect in the fourth development path (RS[1]→PC[3]→MP[2]), as shown in Table 18. The OR aspect can be improved through the RS aspect in the fifth development path (RS[1]→OR[4]→PC[3]→MP[2]), and then the BM aspect can be improved by the OR aspect and the OR aspect can be improved by the RS aspect in the sixth development path (RS[1]→OR[4]→BM[5]→MP[2]). The BM aspect can be improved through the RS aspect in the seventh development path (RS[1]→BM[5]→PC[3]→MP[2]). The BM aspect can be improved by the OR aspect and OR aspect can be improved by the RS aspect in the eight development path (RS[1]→OR[4]→BM[5]→PC[3]→MP[2]) as shown in Table 18.

The ranking of the AI (Accessibility Index) is MP⊃PC⊃RS⊃OR⊃BM, and the RS aspect cannot improve the ME aspect in the first development path (RS[3]→MP[1]). The RS aspect can improve the OR aspect in the second development path (RS[3]→OR[3]→MP[1]), and then the RS aspect can improve the BM aspect in the third development path (RS[3]→BM[5]→MP[1]). The RS aspect cannot improve the PC aspect and PC cannot improve the MP aspect in the fourth development path (RS[3]→PC[2]→MP[1]). The RS aspect can improve the OR aspect in the fifth development path (RS[3]→OR[3]→PC[2]→MP [1]). The RS aspect can improve OR aspect and the OR aspect can improve the BM aspect in the sixth development path (RS[3]→OR[3]→BM[5]→MP [1]). The RS aspect can improve BM aspect in the seventh development path (RS[3]→BM[5]→PC[2]→MP [1]), and then the RS aspect can improve the OR aspect and the OR can improve the BM aspect in the eight development path (RS[3]→OR[3]→BM[5]→PC[2]→MP [1]) as shown in Table 18. Hence, the IAA-NRM approach combines the result of the II (Importance Index) development paths and AI (Accessibility Index) development paths; the suited development paths are shown in Table 18. There are the six suited development paths (RS→OR→MP; RS→BM→MP; RS→OR→PC→MP; RS→OR→BM→MP; RS→BM→PC→MP; RS→OR→BM→PC→MP) for end users, as shown in Table 18.

## 5. Conclusions

Since the COVID-19 pandemic is causing surged demand of medical products and the specific implementation criteria of Med-Tech industry, the main purpose of this study is to analyze the industry entry strategy of the critical development paths for the new investors from three different perspectives. There are five steps of the research structure. The first step is the expert interviews based on literature and related works to provide dimensions of strategy development. The research eventually identified five dimensions, including Professional Capability (PC), Business Management (BM), Operation Resources (OR), Regulation System (RS), and Marketing Promotion (MP). The second step is the survey process, including questionnaire design, data collection, and item reliability test. Then, the third step is using the IAA (Importance Accessibility Analysis) method to confirm the status of the importance and accessibility of the evaluation dimension. Furthermore, the fourth step is to formulate the development path through the NRM (Network Relation Map) method. After that, the last step is to examine the development strategies for industrial entry based on three stakeholders’ perspectives, including vendor, channel, and end-user. There are several research findings as follows.

1. There is one common strategic path from the vendor, channel, and end user’s point of view, from the Regulation System to Operation Resources and then Marketing Promotion (RS→OR→MP).
2. There are two common strategic paths from the vendor and the channel’s perspective. The first one is starting from the Regulation System to Marketing Promotion. The other one is from the Regulation System to Operation Resources and then Marketing Promotion (RS→MP, RS→OR→MP).
3. From vendor and end-user, there are several common strategic paths, including RS→OR→MP, RS→BM→MP, RS→OR→PC→MP, RS→OR→BM→MP, RS→BM→PC→MP, and RS→OR→BM→PC→MP. All these critical paths start with the Regulation System. In the Med-Tech industry, the Regulation System does play a key factor in manufacturing and sales. For any newcomers, the enterprise has to gain a certificate from the Regulation System in order to produce and sell. This is a Med-Tech industry feature different from other industries.
4. From channel and end-user, there is one common critical path, which is from the Regulation System to Operation Resources and then Marketing Promotion (RS→OR→MP).

Since history, people have been hit by pandemics worldwide, such as Severe Acute Respiratory Syndrome (SARS), Middle East Respiratory Syndrome (MERS), or COVID-19. Although the public health system needs to be revitalized based on comprehensive, continuous, and integrated functions [27], improving the urgently needed supply response and quantity in the medical technology industry is also a practical issue. The results show the regulation system plays the most important role in the entry strategy of Med-Tech enterprises. Therefore, the government agency has to be more flexible and have a fast response when a pandemic occurs. For the newcomers, the qualification inspection could be processed with a specified standard or limited production type in order to speed up the procedure. In addition, in the case of surging huge demand for medical supply, the emergency medical inventory level or type needs to be reviewed. Moreover, the Med-Tech company needs to build up a close relationship with their suppliers, such as implementing a supply chain management system, in order to respond to the market demand flexibly and efficiently.

## Figures and Tables

**Table 1:** The descriptions of aspects/criteria for industrial entry consideration of the Med-Tech industry.

Aspects/Criteria	Descriptions
**Professional Capability (PC)**	
Product innovation capability (PC1)	Medical material manufacturers can enhance the company’s operating margin and can effectively, through product innovation capability, reduce and price competition with a homogenization product.
R&D and manufacturing capability (PC2)	Medical material manufacturers can increase the products’ differentiation with competitors by improving research and development capabilities and control manufacturing costs through manufacturing capabilities.
Marketing capability (PC3)	Medical material manufacturers improve their revenue forecasting capabilities by understanding market demand changes and industrial development trends and can deploy organizational resources effectively.
Branding capability (PC4)	Medical material manufacturers can get rid of the low-margin OEM problem by establishing their brands and increasing their business benefits by their brand.
**Business Management (BM)**	
STable cash flow (BM1)	Medical material manufacturers must consider conflicts with existing brand manufacturers when moving in their own brands and need to assess the impact of reduced OEM income.
Motivate all team to transform (BM2)	Company leaders must assess the impact of entry and let members have unity of purpose to help reduce the entry impact.
Merge and acquisition (BM3)	The company must obtain their own lack of resources through mergers and acquisitions in the process of entry and improve the company’s competitiveness through complementary resources.
International operation experience (BM4)	The company improves market sales and operations by understanding various countries’ customs and user habits in international markets.
**Operation Resources (OR)**	
Fundraising (OR1)	Medical material manufacturers want to promote their own branded products to the market by obtaining sufficient resources for brand promotion, channel preparation, and related matters.
Obtain key recourse (OR2)	The medical material manufacturer can successfully sell their products to the market by determining the availability and sources of critical components or raw materials for the production process.
Obtain market information (OR3)	The medical material manufacturer can successfully put their products into the channel by understanding the different market information, including local culture and habits of consumers in the country of sale.
Train multifunction team member (OR4)	The medical material manufacturer from OEM to OBM medical material manufacturer by developing relevant professional sale and marketing, channel establishing, IP and regulatory, and technology integration.
**Regulation System (RS)**	
Approve by international certification (RS1)	The medical material manufacturer who wants to manufacture and sell medical-related products must plan to obtain FDA/CE/GMP and other international medical equipment certification.
Approve by local sales certification (RS2)	The medical material manufacturers sell medical products in the different countries and regions by obtaining the inspection of the competent local authority and get a local sales permit.
Approve by health insurance (RS3)	The medical material manufacturer sells medical products in the different countries and regions by understanding the payment system and insurance system for medical certification in the country.
Apply intellectual property (RS4)	The medical material manufacturer produces medical-related products by carrying out intellectual property rights distribution and obtaining relevant intellectual property protections, such as local patents/trademarks in the country of sale.
**Marketing Promotion (MP)**	
Build up reputation (MP1)	The medical material manufacturer enters the market initially with lack of popularity; they need to spend more time and resources to obtain the approval of the channel and gain consumers’ favor.
Connect channels (MP2)	The medical material manufacturer enters the market initially; they can speed up product sales by learning more about local consumers’ needs and finding the channel trusted by the locals.
Influence by National image (MP3)	The medical material manufacturer enters the market initially; they are affected by the image of their country of production, therefore have to assess for themselves the image of the state of the producing countries.
Understand different culture (MP4)	Because of the differences in culture and habits in different markets, the medical material manufacturer needs to understand the cultural differences of local countries when entering the market.

**Table 2:** The analysis of reliability (Cronbach *α*).

Items	Aspects/Criteria	Alpha	Result
Importance index		0.931	High
Accessibility index		0.907	High
Aspects of evaluation system		0.921	High

Note: Cronbach suggest Alpha α-value: α≤0.35 are low reliability, 0.35<α<0.7 Middle reliability, α≥0.7 is high reliability.

**Table 3:** The Importance Accessibility Analysis (IAA) for industrial entry.

Aspects	ID		AD		(ID, AD)
	MI	SI	MA	SA	
Professional Capability (PC)	8.646	0.860	7.826	0.500	(H, H)
Business Management (BM)	7.682	−1.610	7.338	−1.695	(L, L)
Operation Resources (OR)	8.270	−0.103	7.692	−0.102	(L, L)
Regulation System (RS)	8.626	0.808	7.833	0.535	(H, H)
Marketing Promotion (MP)	8.328	0.045	7.884	0.762	(H, H)
Average	8.311	0.000	7.715	0.000	
Standard deviation	0.390	1.000	0.222	1.000	
Maximum	8.646	0.860	7.884	0.762	
Minimum	7.682	−1.610	7.338	−1.695	

Note 1: (H, H) is the criterion of high importance and high accessibility, (H, L) is the criterion of high importance but low accessibility, (L, L) is the criterion of low importance and low accessibility, and (L, H) is the criterion of a low importance but high accessibility. Note 2: MI, SI, MA, and SA stand for importance value, standardized importance value, accessibility value, and standardized accessibility value, respectively.

**Table 4:** The original average influence matrix (A).

Aspects	PC	BM	OR	RS	MP	Total
Professional Capability (PC)	0.000	2.727	2.747	2.657	2.879	11.010
Business Management (BM)	2.828	0.000	3.010	2.596	2.808	11.242
Operation Resources (OR)	3.030	3.000	0.000	2.596	2.758	11.384
Regulation System (RS)	2.758	2.667	2.616	0.000	2.909	10.949
Marketing Promotion (MP)	2.636	2.848	2.697	2.667	0.000	10.848
Total	11.253	11.242	11.071	10.515	11.354	-

**Table 5:** The direct influence matrix (D).

Aspects	PC	BM	OR	RS	MP	Total
Professional Capability (PC)	0.000	0.240	0.241	0.233	0.253	0.967
Business Management (BM)	0.248	0.000	0.264	0.228	0.247	0.988
Operation Resources (OR)	0.266	0.264	0.000	0.228	0.242	1.000
Regulation System (RS)	0.242	0.234	0.230	0.000	0.256	0.962
Marketing Promotion (MP)	0.232	0.250	0.237	0.234	0.000	0.953
Total	0.988	0.988	0.972	0.924	0.997	-

**Table 6:** The degree of direct influence.

Aspects	Sum of Row	Sum of Column	Sum of Row and Column	Importance of Influence
Professional Capability (PC)	0.967	0.988	1.956	3
Business Management (BM)	0.988	0.988	1.975	1
Operation Resources (OR)	1.000	0.972	1.972	2
Regulation System (RS)	0.962	0.924	1.886	5
Marketing Promotion (MP)	0.953	0.997	1.950	4

**Table 7:** The indirect influence matrix (***ID***).

Aspects	PC	BM	OR	RS	MP	Total
Professional Capability (PC)	7.327	7.284	7.189	6.892	7.335	36.028
Business Management (BM)	7.404	7.457	7.306	7.013	7.463	36.643
Operation Resources (OR)	7.475	7.479	7.437	7.087	7.544	37.022
Regulation System (RS)	7.247	7.253	7.160	6.905	7.300	35.866
Marketing Promotion (MP)	7.200	7.197	7.108	6.813	7.301	35.619
Total	36.653	36.671	36.201	34.710	36.943	-

**Table 8:** The full influence matrix (***T***).

Aspects	PC	BM	OR	RS	MP	*d*
Professional Capability (PC)	7.327	7.524	7.430	7.125	7.588	36.995
Business Management (BM)	7.652	7.457	7.570	7.241	7.710	37.630
Operation Resources (OR)	7.741	7.743	7.437	7.315	7.786	38.022
Regulation System (RS)	7.489	7.487	7.390	6.905	7.556	36.828
Marketing Promotion (MP)	7.432	7.447	7.345	7.047	7.301	36.572
*r*	37.641	37.659	37.173	35.633	37.941	

**Table 9:** The degree of full influence.

Aspects	{*d*}	{*r*}	{*d + r*}	{*d* − *r*}
Professional Capability (PC)	36.995	37.641	74.636	−0.646
Business Management (BM)	37.630	37.659	75.289	−0.029
Operation Resources (OR)	38.022	37.173	75.195	0.849
Regulation System (RS)	36.828	35.633	72.461	1.195
Marketing Promotion (MP)	36.572	37.941	74.513	−1.369

**Table 10:** The net influence matrix for industrial entry.

Aspects	PC	BM	OR	RS	MP
Professional Capability (PC)	-				
Business Management (BM)	0.128	-			
Operation Resources (OR)	0.311	0.174	-		
Regulation System (RS)	0.363	0.246	0.076	-	
Marketing Promotion (MP)	−0.156	−0.263	−0.440	−0.509	-

**Table 11:** The development strategy for urban sustainable development.

Aspects	IAA			NRM			Strategy
	ID	AD	(ID, AD)	*d + r*	*d − r*	(R, D)	
Professional Capability (PC)	0.860	0.500	(H, H)	74.636	−0.646	ID (+,−)	A
Business Management (BM)	−1.610	−1.695	(L, L)	75.289	−0.029	ID (+,−)	C
Operation Resources (OR)	−0.103	−0.102	(L, L)	75.195	0.849	D (+,+)	C
Regulation System (RS)	0.808	0.535	(H, H)	72.461	1.195	D (+,+)	A
Marketing Promotion (MP)	0.045	0.762	(H, H)	74.513	−1.369	ID (+,−)	A

Notes: The development strategies include four types: Development strategy A (direct acquisition), Development strategy B (strategic alliance), Development strategy C (maintain status), and Development strategy D (in house development).

**Table 12:** The suited development paths for industrial entry.

	II (Importance Index)	AI (Accessibility Index)
Rank	PC[1] > RS[2] > MP[3] > OR[4] > BM[5]	MP[1] > RS [2] > PC[3] > OR[4] > BM[5]
Development paths	1. RS[2]→MP [3] {Y} 2. RS[2]→OR[4]→MP[3] {Y} 3. RS[2]→BM[5]→MP[3] {Y} 4. RS[2]→PC[1]→MP[3] {Y} 5. RS[2]→OR[4]→PC[1]→MP[3] {Y} 6. RS[2]→OR[4]→BM[5]→MP[3] {Y} 7. RS[2]→BM[5]→PC[1]→MP[3] {Y} 8. RS[2]→OR[4]→BM[5]→PC[1]→MP[3] {Y}	1. RS[2]→MP[1] {N} 2. RS[2]→OR[4]→MP[1] {Y} 3. RS[2]→BM[5]→MP[1] {Y} 4. RS[2]→PC[3]→MP[1] {Y} 5. RS[2]→OR[4]→PC[3]→MP[1] {Y} 6. RS[2]→OR[4]→BM[5]→MP[1] {Y} 7. RS[2]→BM[5]→PC[3]→MP[1] {Y} 8. RS[2]→OR[4]→BM[5]→PC[3]→MP[1] {Y}
Suited development paths	2. RS→OR→MP 3. RS→BM→MP 4. RS→PC→MP 5. RS→OR→PC→MP 6. RS→OR→BM→MP 7. RS→BM→PC→MP 8. RS→OR→BM→PC→MP	

**Table 13:** The development strategy for vendors.

Aspects	IAA			NRM			Strategy
Venders	ID	AD	(ID, AD)	*D + r*	*d − r*	(R, D)	
Professional Capability (PC)	1.165	0.800	(H, H)	74.636	−0.646	ID (+,−)	A
Business Management (BM)	−1.456	−1.656	(L, L)	75.289	−0.029	ID (+,−)	C
Operation Resources (OR)	−0.185	−0.112	(L, L)	75.195	0.849	D (+,+)	C
Regulation System (RS)	0.671	0.744	(H, H)	72.461	1.195	D (+,+)	A
Marketing Promotion (MP)	−0.196	0.223	(L, H)	74.513	−1.369	ID (+,−)	B

Notes: The development strategies include four types: development strategy A (direct acquisition), development strategy B (strategic alliance), development strategy C (maintain status), and development strategy D (in house development).

**Table 14:** The development strategy for channels.

Aspects	IAA			NRM			Strategy
Channel	II	AI	(II, AI)	*d + r*	*d − r*	(R, D)	
Professional Capability (PC)	0.148	−0.970	(H, L)	74.636	−0.646	ID (+,−)	D
Business Management (BM)	−1.467	−1.174	(L, L)	75.289	−0.029	ID (+,−)	C
Operation Resources (OR)	−0.083	0.511	(L, H)	75.195	0.849	D (+,+)	B
Regulation System (RS)	1.347	1.021	(H, H)	72.461	1.195	D (+,+)	A
Marketing Promotion (MP)	0.055	0.613	(H, H)	74.513	−1.369	ID (+,−)	A

Notes: The development strategies include four types: development strategy A (direct acquisition), development strategy B (strategic alliance), development strategy C (maintain status), and development strategy D (in house development).

**Table 15:** The development strategy for end users.

Aspects	IAA			NRM			Strategy
End users	ID	AD	(ID, AD)	*d + r*	*d − r*	(R, D)	
Professional Capability (PC)	0.339	0.636	(H, H)	74.636	−0.646	ID (+,−)	A
Business Management (BM)	−1.739	−1.177	(L, L)	75.289	−0.029	ID (+,−)	C
Operation Resources (OR)	0.100	−0.413	(H, L)	75.195	0.849	D (+,+)	D
Regulation System (RS)	0.698	−0.413	(H, L)	72.461	1.195	D (+,+)	D
Marketing Promotion (MP)	0.602	1.367	(H, H)	74.513	−1.369	ID (+,−)	A

Notes: The development strategies include four types: development strategy A (direct acquisition), development strategy B (strategic alliance), development strategy C (maintain status), and development strategy D (in house development).

**Table 16:** The suited development paths for vendors.

	II (Importance Index)	AI (Accessibility Index)
Rank	PC[1] > RS[2] > OR[3] > MP[4] > BM[5]	PC[1] > RS[2] > MP[3] > OR[4] > BM[5]
Development paths	1. RS[2]→MP[4] {Y} 2. RS[2]→OR[3]→MP[4] {Y} 3. RS[2]→BM[5]→MP[4] {Y} 4. RS[2]→PC[1]→MP[4] {Y} 5. RS[2]→OR[3]→PC[1]→MP[4] {Y} 6. RS[2]→OR[3]→BM[5]→MP[4] {Y} 7. RS[2]→BM[5]→PC[1]→MP[4] {Y} 8. RS[2]→OR[3]→BM[5]→PC[1]→MP[4] {Y}	1. RS[2]→MP[3] {Y} 2. RS[2]→OR[4]→MP[3] {Y} 3. RS[2]→BM[5]→MP[3] {Y} 4. RS[2]→PC[1]→MP[3] {Y} 5. RS[2]→OR[4]→PC[1]→MP[3] {Y} 6. RS[2]→OR[4]→BM[5]→MP[3] {Y} 7. RS[2]→BM[5]→PC[1]→MP[3] {Y} 8. RS[2]→CR[4]→BM[5]→PC[1]→MP[3] {Y}
Suited development paths	1. RS→MP 2. RS→OR→MP 3. RS→BM→MP 4. RS→PC→MP 5. RS→OR→PC→MP 6. RS→OR→BM→MP 7. RS→BM→PC→MP 8. RS→OR→BM→PC→MP	

**Table 17:** The suited development paths for channels.

	II (Importance Index)	AI (Accessibility Index)
Rank	RS[1] > PC[2] > MP[3] > OR[4] > BM[5]	RS[1] > ME[2] > OR[3] > PC[4] > OM[5]
Development paths	1. RS[1]→MP[3] {Y} 2. RS[1]→OR[4]→MP[3] {Y} 3. RS[1]→BM[5]→MP[3] {Y} 4. RS[1]→PC[2]→MP[3] {Y} 5. RS[1]→OR[4]→PC[2]→MP[3] {Y} 6. RS[1]→OR[4]→BM[5]→MP[3] {Y} 7. RS[1]→BM[5]→PC[2]→MP[3] {Y} 8. RS[1]→OR[4]→BM[5]→PC[2]→MP[3] {Y}	1. RS[1]→ME[2] {Y} 2. RS[1]→OR[3]→ME[2] {Y} 3. RS[1]→OM[5]→ME[2] {Y} 4. RS[1]→PC[4]→ME[2] {Y} 5. RS[1]→OR[3]→PC[4]→ME[2] {Y} 6. RS[1]→OR[3]→OM[5]→ME[2] {Y} 7. RS[1]→OM[5]→PC[4]→ME[2] {Y} 8. RS[1]→OR[3]→OM[5]→PC[4]→ME[2] {Y}
Suited development paths	1. RS→MP 2. RS→OR→MP 3. RS→BM→MP 4. RS→PC→MP 5. RS→OR→PC→MP 6. RS→OR→BM→MP 7. RS→BM→PC→MP 8. RS→OR→BM→PC→MP	

**Table 18:** The suited development paths for end users.

	II (Importance Index)	AI (Accessibility Index)
Rank	RS[1] > MP[2] > PC[3] > OR[4] > BM[5]	MP[1] > PC[2] > RS[3] = OR[3] > BM[5]
Development paths	1. RS[1]→MP[2] {Y} 2. RS[1]→OR[4]→MP[2] {Y} 3. RS[1]→BM[5]→MP[2] {Y} 4. RS[1]→PC[3]→MP[2] {Y} 5. RS[1]→OR[4]→PC[3]→MP[2] {Y} 6. RS[1]→OR[4]→BM[5]→MP[2] {Y} 7. RS[1]→BM[5]→PC[3]→MP[2] {Y} 8. RS[1]→OR[4]→BM[5]→PC[3]→MP[2] {Y}	1. RS[3]→MP[1] {N} 2. RS[3]→OR[3]→MP[1] {Y} 3. RS[3]→BM[5]→MP[1] {Y} 4. RS[3]→PC[2]→MP[1] {N} 5. RS[3]→OR[3]→PC[2]→MP [1] {Y} 6. RS[3]→OR[3]→BM[5]→MP [1] {Y} 7. RS[3]→BM[5]→PC[2]→MP [1] {Y} 8. RS[3]→OR[3]→BM[5]→PC[2]→MP [1] {Y}
Suited development paths	2. RS→OR→MP 3. RS→BM→MP 5. RS→OR→PC→MP 6. RS→OR→BM→MP 7. RS→BM→PC→MP 8. RS→OR→BM→PC→MP	
